# Differentially Regulated miRNAs and Their Related Molecular Pathways in Lichen Sclerosus

**DOI:** 10.3390/cells10092291

**Published:** 2021-09-02

**Authors:** Xiaohui Tan, Shuyang Ren, Canyuan Yang, Shuchang Ren, Melinda Z. Fu, Amelia R. Goldstein, Xuelan Li, Leia Mitchell, Jill M. Krapf, Charles J. Macri, Andrew T. Goldstein, Sidney W. Fu

**Affiliations:** 1 Departments of Medicine (Division of Genomic Medicine), and of Microbiology, Immunology and Tropical Medicine, The George Washington University School of Medicine and Health Sciences, 2300 Eye Street, N.W., Ross Hall 402C, Washington, DC 20037, USA; janexht@gwu.edu (X.T.); shuyangren@gwmail.gwu.edu (S.R.); canyuanyang@gwmail.gwu.edu (C.Y.); sren2@gwmail.gwu.edu (S.R.); melfu@gwmail.gwu.edu (M.Z.F.); 2 Department of Biology, Duke University, Durham, NC 27708, USA; amelia.goldstein@duke.edu; 3 Department of OB/GYN, The First Affiliated Hospital, Xi’an Jiaotong University, Xi’an 710061, China; lixuelan1225@126.com; 4 The Center for Vulvovaginal Disorders, Washington, DC 20037, USA; leiamitchell11@gmail.com (L.M.); jillkrapf@gmail.com (J.M.K.); cmacri@mfa.gwu.edu (C.J.M.); 5 Department of OB/GYN, The George Washington University School of Medicine and Health Sciences, Washington, DC 20037, USA

**Keywords:** lichen sclerosus, miRNA, gene network, pathogenesis

## Abstract

Lichen sclerosus (LS) is a chronic inflammatory skin disorder with unknown pathogenesis. The aberrant expression of microRNAs (miRNAs) is considered to exert a crucial role in LS. We used the next-generation sequencing technology (RNASeq) for miRNA profiling and Ingenuity Pathway Analysis (IPA) for molecular network analysis. We performed qRT-PCR, miRNA transfection and Matrigel assays for functional studies. We identified a total of 170 differentially expressed miRNAs between female LS and matched adjacent normal tissue using RNASeq, with 119 upregulated and 51 downregulated. Bioinformatics analysis revealed molecular networks that may shed light on the pathogenesis of LS. We verified the expression of a set of miRNAs that are related to autoimmunity, such as upregulated miR-326, miR-142-5p, miR-155 and downregulated miR-664a-3p and miR-181a-3p in LS tissue compared to the matched adjacent normal tissue. The differentially expressed miRNAs were also verified in blood samples from LS patients compared to healthy female volunteers. Functional studies demonstrated that a forced expression of miR-142-5p in human dermal fibroblast PCS-201-010 cells resulted in decreased cell proliferation and migration. These findings suggest that differentially expressed miRNAs may play an important role in LS pathogenesis; therefore, they could serve as biomarkers for LS management.

## 1. Introduction

Lichen sclerosus (LS) is the condition of chronic, lymphocyte-mediated inflammatory dermatitis that has a negative impact on quality of life and may proceed to malignant disease [1,2]. LS can affect any part of the skin, but it most frequently occurs in the anogenital area, with a higher incidence of onset among premenarchal and post-menopausal females [3,4]. The typical lesions of vulvar LS are white plaques and papules, often with areas of ecchymosis, excoriation, and ulceration. Presenting symptoms may include intense pruritus, pain, burning and dyspareunia. Between 4 and 7% of women with LS develop vulvar squamous carcinoma [5]. LS is among one of the most common referrals for vulvar pruritus and is the most common cause of inflammatory structural changes to the vulvar region [6,7,8]. Histologically, LS is characterized by a band-like lymphocytic infiltrate and a thinned epidermis with vacuolar changes in the basal layer. In the long-standing, classic LS, the lymphocytic infiltrate is located under a band of homogenized collagen below the dermoepidermal junction [9]. Although there is increasing evidence that autoimmune mechanisms play a pathogenic role, the etiology of LS has not yet been adequately explained [10,11,12]. Currently, LS diagnosis is based on the physical examination and dermatopathological analysis of skin biopsy. However, there is a lack of unanimity, especially in the early stages of disease. In addition, the histological evaluation of LS and other immunologically mediated diseases, such as erosive lichen planus (ELP), can be difficult, as the first phase of LS can show histological overlap with ELP [13]. The therapeutic options for LS, namely ultrapotent topical corticosteroids, require long-term or life-long regimens and are not curative [14,15]. Therefore, the development of non-invasive and reliable biomarkers could potentially impact LS screening, diagnosis and treatment. 

MicroRNAs (miRNAs) are small, endogenous RNA molecules that act as regulators of gene expression by binding to the 3′-untranslated region (3′ UTR) of target mRNAs [16,17]. They play critical roles in cellular homeostasis and biological processes, and are therefore involved in the development of various diseases in broad pathological conditions [18]. Accumulating evidence suggests that dysregulation of miRNAs is associated with inflammation and autoimmunity [19], suggesting their roles in LS development. For example, miR-142 overexpression might be involved in the autoimmune neuroinflammation and pathogenesis of multiple sclerosis through changing the pattern of T cell differentiation [20]. The downregulation of miR-142-3p inhibits keratinocyte proliferation in oral lichen planus (OLP) [21]. Although the aberrant expression of miRNAs has been implicated in LS pathogenesis [12,22], there are very limited studies to date. 

The aim of this study was to explore the role of miRNAs in vulvar LS pathogenesis by identifying differentially expressed miRNAs and their associated molecular networks and decipher the functional role of selected candidate miRNAs for their potential clinical utility in the diagnosis and treatment of LS.

## 2. Materials and Methods

### 2.1. Clinical Samples

With The George Washington University’s IRB approval and patients’ consent, 4 mm vulvar punch biopsies of active LS along with matched normal adjacent tissues, and 2 mL of peripheral blood samples were collected at the time of clinical consultation (Table 1). Subjects with vulvar LS were diagnosed by both visual observation and dermatopathological confirmation, and recruited from a gynecology practice specializing in vulvar dermatoses. Tissue and blood samples were immediately placed into RNAlater Storage Solution (Invitrogen AM7020) before being transported to the research lab for storage in a −80 °C freezer or immediate RNA isolation. An additional 23 peripheral blood samples from healthy female volunteers were used as controls.

### 2.2. Cell Lines

Normal human dermal fibroblast, PCS-201-010, was purchased from ATCC and cultured in Fibroblast Growth Kit—Serum-Free (ATCC^®^ PCS-201-040) with 3.75% L-glutamine, 0.1% hydrocortisone hemisuccinate, 0.25% HLL supplement, 0.1% rh FGF basic, 0.1% TGF β-1 supplement, 0.1% rh insulin, and 0.1% ascorbic acid. The cell line was cultured at 37 °C in a humidified incubator with 5% CO_2_.

### 2.3. RNA Extraction, RNA Library Preparation and RNASeq Assays

Total RNA was isolated from cell line, LS and the matched adjacent normal tissue using the TRIzol reagent (Cat#15596–026, Invitrogen, Carlsbad, CA, USA). Blood RNA was isolated by the RiboPure™ RNA Purification Kit (Cat#AM1928, Life Technologies, Frederick, MD, USA). The total RNA from LS and the matched adjacent normal tissue was subsequently purified with the miRNeasy Mini kit (Cat#217004, Qiagen, Germantown, MD, USA) according to the manufacturer’s instructions. RNA quality, quantity, and purity were determined using a Bioanalyzer 2100 and RNA 6000 Nano LabChip Kit (Cat# 5067-1511, Agilent, Foster City, CA, USA). For RNA sequencing, 1 μg of the total RNA was used to prepare small a RNA library using TruSeq Small RNA Sample Prep Kits (Illumina, San Diego, CA, USA). miRNA deep sequencing (RNASeq) was performed by LC Sciences (Houston, TX, USA) using Illumina Hiseq 4000 Sequencer (Illumina, San Diego, CA, USA). Raw sequencing reads were obtained using the Illumina’s Sequencing Control Studio software (v2.8; Illumina, Inc. San Diego, CA, USA) (https://www.lcsciences.com/documents/sample_data/microrna_sequencing/miRNA_sequencing_report_DEMO.html, accessed on 21 March 2019).

### 2.4. Data Preprocessing

Preliminary quality control analysis was performed by removing adapter dimers, junk, low complexity, common RNA families such as snoRNA snRNA, rRNA and tRNA, and repeats sequences from raw sequence reads obtained from sequencing using the ACGT101-miR program (LC Sciences, Houston, TX, USA). Only unique sequences of 18–26 nucleotides (nt) in length were retained and compared to known homo sapiens miRNAs in miRBase 21.0 by BLAST search. Length variation at both the 3′ and 5′ ends and one mismatch inside of the sequence were allowed in the alignment. The unique sequences mapping to human mature miRNAs in hairpin arms were identified as known miRNAs. The unique sequences mapping to the other arm of known specific species precursor hairpin, opposite the annotated mature miRNA-containing arm, were considered to be novel 5p- or 3p-derived miRNA candidates. The remaining sequences were mapped to other selected precursors in miRBase 21.0 by BLAST search, and the mapped pre-miRNAs were further BLASTed against the human genomes to determine their genomic locations. 

### 2.5. Identification of Differentially Expressed miRNAs 

The quantitative miRNAs data were normalized to reads per million (RPM), which was calculated as RPM = miRNA counts/total counts of each sample × 1,000,000, and then log 2-transformed. The differently expressed miRNAs between LS and the matched normal tissues were identified using the paired Student’s *t*-test. miRNAs were considered to be differentially expressed at *p* < 0.05, fold change (FC) >1.5 or <1/1.5 and average expression > (log2) 5 RPM. To determine whether the differentially expressed miRNAs were able to differentiate LS from the normal control, clustering analysis was performed to generate a heat map using the hclust function implemented in the R package (v2.14.1; http://www.R-project.org, accessed on 20 April 2020), which uses the Euclidean distance and Ward’s method.

### 2.6. Pathway Analysis

The differentially expressed miRNAs were subjected to molecular pathway analysis using the Ingenuity Pathway Analysis (IPA) software. Focus miRNA genes were identified as having direct interactions with other genes in the database. Pathways of highly interconnected miRNAs and their target genes were identified by statistical likelihood (*p* < 0.05). The networks were generated through the use of IPA (QIAGEN, Germantown, MD, USA) (https://www.qiagenbioinformatics.com/products/ingenuity-pathway-analysis, accessed on 5 June 2021).

### 2.7. Quantitative Real-Time Reverse Transcription-PCR (qRT-PCR)

To verify miRNA sequencing results, miRNA expression was assayed by quantitative reverse transcriptase-polymerase chain reaction (qRT-PCR) using the Taqman MiRNA Reverse Transcript Kit (Cat# 4366596, Thermo Fisher, Waltham, MA, USA). SYBR Green-based qRT-PCR was used to measure the relative expression of selected target genes. Five hundred nanograms of the total RNA were reverse transcribed to generate complementary DNA (cDNA) employing the High Capacity cDNA Reverse Transcript Kit (Thermo Scientific, Rockford, IL, USA). The QuantStudio™ 3 Real-Time PCR System (ABI) was used for these assays. Relative expression was calculated using the 2−_ΔΔ_CT method [23].

### 2.8. Target Gene Prediction

To determine the function of a set of selected miRNAs in LS pathogenesis, we performed target prediction using miRDB, DIANA and TargetScan databases [24,25,26,27,28]. Target genes of interest were further evaluated by literature search focusing on their involvement in LS linked inflammatory pathways.

### 2.9. miRNA Precursors and Plasmid Transfection

miRNA transient-transfection was performed as described [23,29]. Briefly, the miRNA precursors (miR-142-5p mimic, inhibitor and mock control) were transiently transfected into the normal human dermal fibroblast, PCS-201-010 cell line by the Lipofectamine RNAiMAX (Life Technologies) using the Opti-MEM I Reduced Serum Medium (Life Technologies). Cells were subjected to further analysis after 24 h, 48 h and 72 h post-transfection.

### 2.10. Matrigel Invasion Assays

Matrigel invasion assays were performed using the BD BioCoat™ Matrigel™ Invasion Chamber (Cat# 354480, BD Biosciences, San Jose, CA, USA) as previously described [23,30]. Briefly, prior to the start of each experiment, 500 μL of warm (37 °C) serum-free DMEM medium was added to the upper and lower chambers and allowed to rehydrate for 2 h in a 37 °C cell culture incubator, while 8 × 10^4^ cells were transfected by either miR-142-5p mimic or an inhibitor with the mock controls for 24 h and seeded onto the top chamber of pre-wetted inserts. Cells were incubated in a Matrigel chamber in a 37 °C humidified incubator with 5% CO_2_ for 24 h. The invasive cells present were fixed, stained with the Diff-Quick staining solution and counted (five microscope fields under the 10× lens). Experiments were done in duplicates for each cell line twice. Cell counts were performed on five non-overlapping random fields for each chamber and four chambers were counted for each experimental point, with the percentage of invasive cells being normalized to corresponding controls. 

### 2.11. Statistical Analysis 

Statistical analysis for the differentially expressed miRNAs by RNASeq was described above. All other data are distributed approximately normally by histogram analysis. Data are expressed as the mean ± standard deviation. Differences between groups were determined by Student’s *t*-test or pair *t*-test as appropriate. *p*-value < 0.05 was considered statistically significant.

## 3. Results

### 3.1. miRNA Profiles in LS and Matched Adjacent Tissue

**Figure 1:**
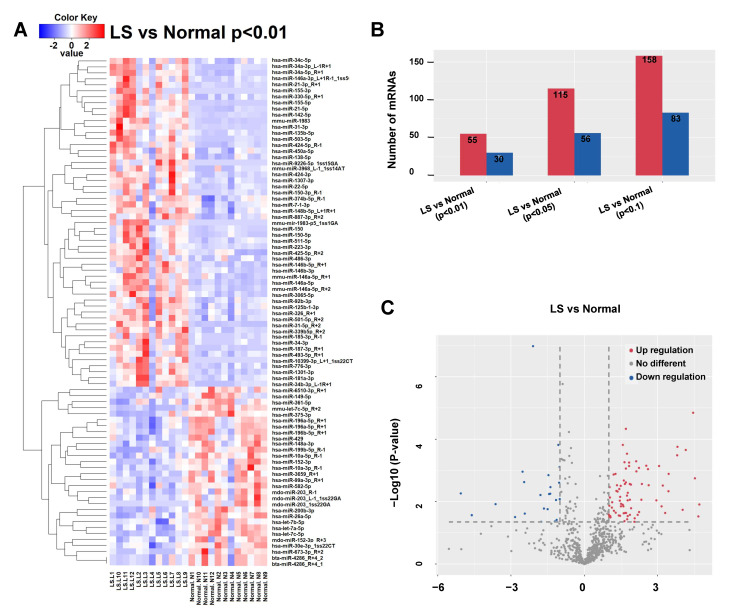
Differentially expressed microRNAs in LS tissue. Red signal, high relative expression; blue signal, low relative expression. (**A**) Clustering analysis of differentially expressed miRNAs between LS and matched adjacent normal tissue. The inclusion criteria were a 2-fold-change in either direction with a *p*-value < 0.01. (**B**) The numbers of differentially expressed miRNAs with 2-fold change under different *p* values (0.01, 0.05 and 0.10). (**C**) The volcano plot revealed more upregulated miRNAs than downregulated ones in LS. The inclusion criteria were a 2-fold-change in either direction with a *p*-value < 0.05.

Twelve pairs of LS and their matched adjacent normal tissue derived from active LS patients were used to conduct the RNASeq assay (Table 1). A total of 781 differentially expressed miRNAs were identified (Supplementary Table S1). Using the Benjamini–Hochberg False Discovery Rate (FDR) multiple testing correction (*p* (corr) < 0.05) method and 1.5-fold change cut-off, we identified 170 miRNAs differentially expressed between LS and normal tissue, including 119 upregulated and 51 downregulated miRNAs (Figure 1).

**Figure 2:**
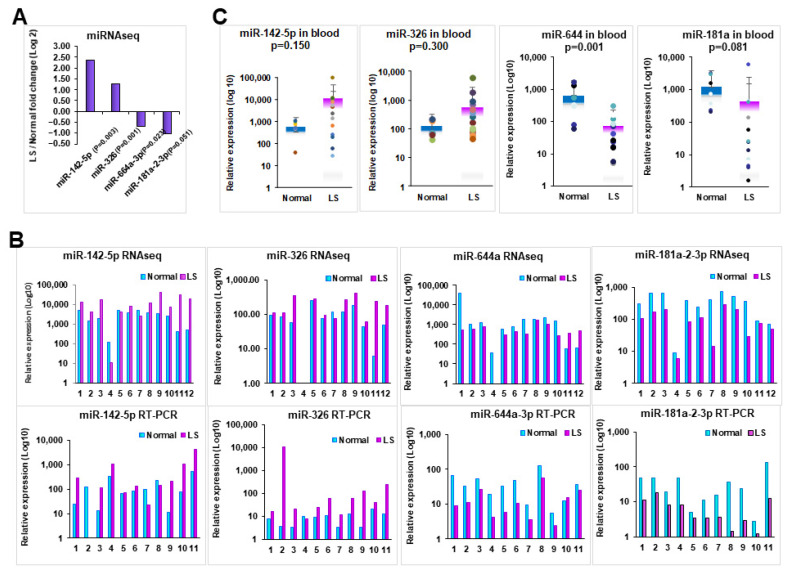
Expression analysis of selected candidate miRNAs in tissue and blood samples. (**A**) Expression of represented miRNAs by RNASeq analysis. (**B**) Expression comparison of dysregulated miRNAs between by RNASeq and qRT-PCR. (**C**) Expression of representative miRNAs in blood samples from patients with LS compared with healthy female volunteers. *p*-values were calculated for statistical significance as indicated for each comparison.

We subsequently verified the expression levels of a set of selected candidate miRNAs that are related to allergic and autoimmune diseases, such as upregulated miRNAs (miR-326, miR-142-5p) and downregulated miRNAs (miR-644a, miR-181a2-3p) [31,32,33], by qRT-PCR in LS tissues from the same patients examined by RNASeq (Figure 2A) and from an additional cohort of LS patients by qRT-PCR. Our data showed that the upregulated miRNAs, miR-326, miR-142-5p and downregulated miRNAs, miR-644a, miR-181a-2-3p were concordant with our RNASeq data (Figure 2B). The full dataset is available upon request.

### 3.2. Verifying the Dysregulation of miRNAs in Peripheral Blood

As an attempt to establish whether miRNA profiling could be used as biomarker for LS, we investigated whether there is a similar miRNA expression pattern in peripheral blood samples. To address this, we examined the miRNA expression in 33 peripheral blood samples including 12 patients subjected to RNASeq, and 21 additional LS patients (Table 1). We found that significantly increased miR-326, miR-142-5p and decreased miR-644a, miR-181a-2-3p in blood samples from LS patients compared to the healthy female volunteers (Figure 2C), which is consistent with the RNASeq data we obtained from tissue samples. This suggests that miRNAs may serve as potential biomarkers for LS diagnosis and treatment.

### 3.3. Gene Network and Pathway Analysis

**Figure 3:**
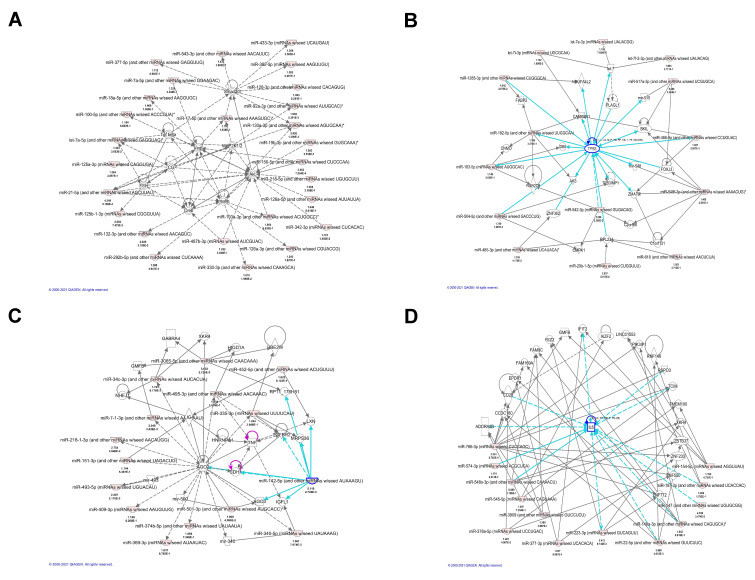
Top representative molecular networks involving differentially expressed miRNAs in LS identified by IPA analysis. (**A)** Differentially expressed miRNAs that are involved in AKT gene regulation. (**B**) Differentially expressed miRNAs that are involved in TP53 regulation. (**C**) Top representative network involving miR-142-5p in cell–cell interaction, cell death, cell survival and cellular assembly and organization0. (**D**) A network involving IL-6 regulation related to inflammatory diseases, organismal injury and abnormalities.

To gain insights about the broader biological context in which the discovered miRNAs operate, we performed network analysis using the significantly differentially expressed miRNAs. The analysis revealed 14 molecular networks enriched for or targeted by miRNAs highly involved in cancer–injury–reproductive diseases, inflammatory diseases, cell morphology and infectious diseases, cell–cell interaction, as well as cell death and survival. Several miRNAs, such as miR-487b-3p, miR-342-3p, miR-218-5p, and miR-126a-5p, were found to be targeting the AKT gene involved in cancer–injury–reproductive diseases (Figure 3A). Other miRNAs targeted genes involved in cell–cell interaction, cell death, cell survival and cellular assembly and organization network including miR-142-5p, miR-151-3p, miR-218-1-3p, miR-335, and miR-340 related with AGO2, AGO3, CDH1, GABRA3 (Figure 3C). These results are similar to previous reports in renal cell carcinoma [34] and thyroid cancer [35]. The results of network analysis are presented in Figure 3. 

### 3.4. Forced Expression of miR-142-5p in Human Dermal Fibroblast PCS-201-010 Resulted in Decreased Cell Migration

**Figure 4:**
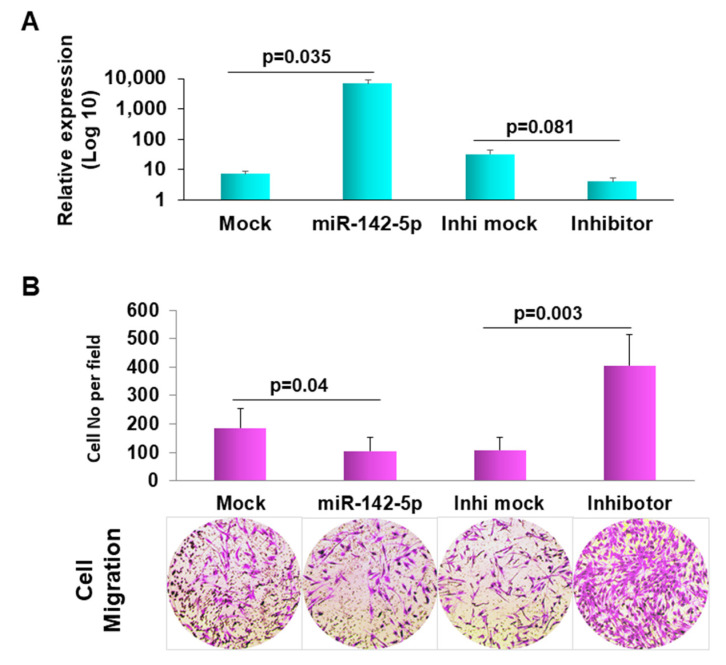
Forced expression of miR-142-5p reduced cell migration. (**A**) Confirmation of transfection of miR-142-5p, inhibitor and the mock controls in normal human dermal fibroblast, PCS-201-010 by qRT-PCR. (**B**) matrigel invasion images (representative ones at the bottom) were pixelized using the Photoshop software version 19.1.3. The migration ability of the cells was displayed as a percentage of the absolute cell numbers. Results are displayed as mean data ± SE (*p* < 0.05). Five fields of unit area on each membrane or whole membrane were counted for cell numbers, and the experiments were repeated three times with triplicates.

miR-142-5p has been reported as a critical modulator of immune cells as well as inflammatory and immunological responses [20,36]. Pathway analysis showed that miR-142-5p involved in cell–cell interaction and the cell death and survival network, suggesting its essential role in LS development. To demonstrate the function of miR-142-5p in LS, we transfected miR-142-5p into human dermal fibroblast PCS-201-010 cells. Since a migrating capability change is essential in fibrogenic disease [37], we measured the migration ability by Matrigel assay. As presented in Figure 4, the forced expression of miR-142-5p exhibited the remarkable inhibition of cell migration compared to the mock transfection, suggesting that miR-142-5p may be involved in the pathogenesis of LS. 

### 3.5. Target Gene Prediction and Verification

Bioinformatics tools were used to predict the potential target genes of hsa-miR-142-5p. For the primary screening, three databases were used including TargetScan (http://www.targetscan.org, accessed on 6 April 2019), miRDB (http://mirdb.org/, accessed on 6 April 2019) and DIANA Tools (http://diana.imis.athena-innovation.gr, accessed on 6 April 2019) to obtain a dataset of potential downstream target genes of miR-142-5p [38,39,40,41]. Seventeen potential target genes (PPARGC1B, RNF165, PAPPA, SLC22A23, CALM1, HDLBP, GAS7, SREBF1, TMOD1, TCF21, HSD11B1, DACH1, ABHD5, RNF128, CADM3, RBM24, DACH1, and LPP) were predicted. The expression level of these genes was assessed after transfection of miR-142-5p.

**Figure 5:**
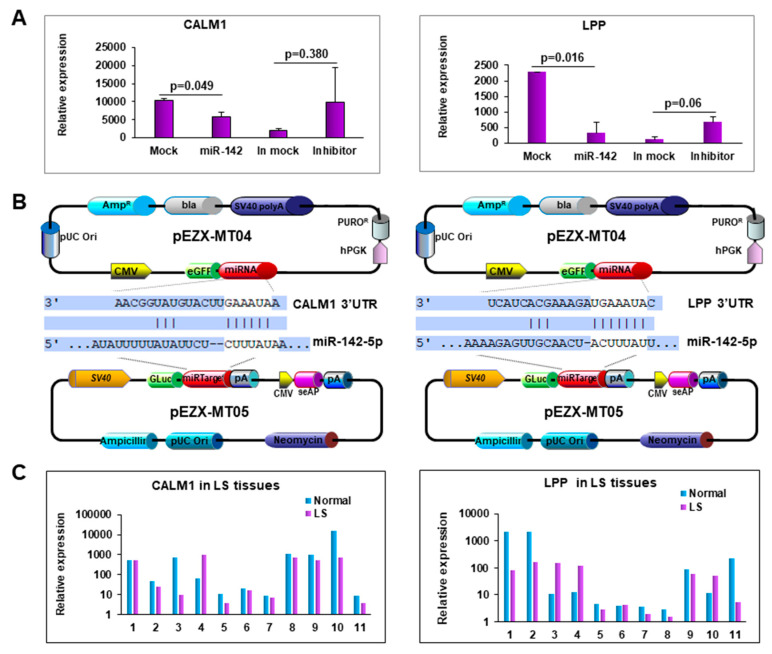
Forced expression of miR-142-5p reduced CALM1 and LPP expression. (**A**) Expression of CALM1 and LPP when miR-142-5p was either overexpressed or knocked-down. (**B**) Map of the plasmids, pEZX-MT04 containing miR-142-5p, and pEZX-MT05 containing 3′-UTR of CALM1 or LPP to illustrate the binding site of miR-1425p to its target genes. (**C**) Expression of CALM1 and LPP in LS tissue.

To verify the effect of miR-142-5p on the predicted target gene, we transfected miR-142-5p into the PCS-201-010 cell line and measured the expression of the predicted genes. Among the predicted genes, CALM1 and LPP were significantly downregulated in miR-142-5p transfected cells compared to the mock transfected ones, while they were upregulated in miR-142-5p inhibitor transfected cells compared to the inhibitor mock transfected ones (Figure 5A,B). To determine miR-142-5p target gene expression in LS tissue, we performed qRT-PCR in 11 paired samples. As expected, we detected a low expression level of CALM1 and LPP in LS compared to their matched adjacent normal tissues (Figure 5C).

## 4. Discussion

We hypothesized that miRNAs could be promising biomarkers for LS diagnosis and treatment based on the following premises: firstly, that miRNA expression is frequently dysregulated in human diseases [42]; secondly, that miRNAs appear to be tissue-specific [43] and thirdly, that miRNAs are exceptionally stable in tissue and blood [44]. To date, no systematic studies aiming to analyze miRNA expression profiles in vulvar LS have been reported. Here, we assessed these miRNA alterations for the first time in a cohort from LS tissue and blood samples in order to identify miRNA biomarker(s) for LS diagnosis and treatment. We identified 170 dysregulated miRNAs, including 119 upregulated miRNAs and 51 downregulated miRNAs in LS. We selected miRNA candidates to further verify their expression in LS by qRT-PCR. Through transcriptome analysis, functional and pathway analyses, we showed that dysregulated miRNAs may play an essential role in LS development through a number of molecular networks. Our IPA analysis revealed 10 top molecular networks (Supplementary Table S2) enriched for or targeted by miRNAs that are highly likely to be involved in cancer–injury–reproductive diseases, inflammatory diseases, cell morphology infections disease, cell–cell interaction and cell death and survival (Figure 3), which may be involved in LS pathogenesis.

miR-155 has numerous functions, including lymphocyte homeostasis maintenance, inflammation and immune system regulation [45]. Increased levels of miR-155 have been found in various inflammatory diseases [46,47,48], including oral lichen planus (OLP) [49] and LS [50]. An increasing number of studies [51,52] have focused on signaling pathways and cytokine release regulated by miR-155. The expression of miR-155 is usually correlated with increased cytokine release, including IFN-γ [53]. Our RNASeq data confirmed that miR-155 was overexpressed in LS using the adjacent normal tissue from the same patients as controls, while Terlou et al. [54] reported similar findings by microarray analysis when compared with normal volunteers. We further confirmed that miR-155 was upregulated in blood samples from LS patients compared to healthy female volunteers. miR-155 targets signaling pathways that are involved in cellular phenotypes which could play an important role in LS development.

Of the differentially expressed miRNAs, we further focused on one of the most significantly dysregulated miRNAs, miR-142-5p. We found that miR-142-5p expression was increased in both the tissue and blood from LS patients. The miR-142 gene is located on chromosome 17 and is broadly conserved between different species [55]. miR-142-5p was noted to be aberrantly expressed in pathological conditions such as cancer, immunologically related disorders, small bowel inflammation, renal fibrosis where inflammation occurs and in biopsies from renal transplant patients with acute rejection [56]. miR-142-5p has been found to regulate macrophage profibrogenic gene expression in chronic inflammation [57] and is overexpressed in oral lichen planus, a chronic T cell mediated inflammatory disease [58]. Two target genes of miR-142-5p, CALM1 and LPP, were downregulated in LS tissue samples. Gene Ontology (GO) annotations indicate that CALM1 is involved in calcium ion binding and protein domain specific binding [59]. CALM1 stimulates protein kinases and phosphatases, and is involved in a molecular pathway that regulate the centrosome cycle and progression through cytokinesis [60]. LPP, a transcriptional co-activator, localizes to the cell periphery, which involves cell–cell adhesion and cell motility [61]. LPP often appears at the junction of chromosomal translocations, resulting in chimeric proteins that may promote pathogenesis, such as tumor growth and other disorders [62]. LPP also plays a structural and transcriptional role at sites of cell adhesion in maintaining cell shape and motility [63], which may be crucial for LS development and progression. These studies suggest that miR-142-5p may be an essential player in LS, and may serve as a potential target for LS management.

## 5. Conclusions

Differentially expressed miRNAs and their related molecular networks may shed light on the pathogenesis of LS. Candidate miRNAs, such as miR-155 and miR-142-5p may serve as diagnostic and/or therapeutic biomarkers for LS.

## Figures and Tables

**Table 1:** Clinical samples of tissue and blood from patients with active lichen sclerosus.

Patient	Tissue	Blood
1 *	Normal + LS	Yes
2 *	Normal + LS	Yes
3	N/A	Yes
4 *	Normal + LS	Yes
5	N/A	Yes
6	N/A	Yes
7 *	Normal + LS	Yes
8	N/A	Yes
9	N/A	Yes
10	N/A	Yes
11	N/A	Yes
12 *	Normal + LS	Yes
13	N/A	Yes
14	N/A	Yes
15 *	Normal + LS	Yes
16 *	Normal + LS	Yes
17 *	Normal + LS	Yes
18	N/A	Yes
19	N/A	Yes
20 *	Normal + LS	Yes
21 *	Normal + LS	Yes
22	N/A	Yes
23 *	Normal + LS	Yes
24 *	Normal + LS	Yes
25	Normal + LS	Yes
26	N/A	Yes
27	Normal + LS	Yes
28	N/A	Yes
29	N/A	Yes
30	Normal + LS	Yes
31	Normal + LS	Yes
32	Normal + LS	Yes
33	Normal + LS	Yes

* Samples used for RNASeq analysis. N/A, not available.

## Data Availability

The RNASeq data will be deposited into GEO database.

## Supplementary Materials

The following are available online at https://www.mdpi.com/article/10.3390/cells10092291/s1, Table S1: A complete list of differentially expressed miRNAs in LS; Table S2: IPA analysis network list (top 10).
