# Supplementary material for: Differentially Regulated miRNAs and Their Related Molecular Pathways in Lichen Sclerosus
Source: Cells. 2021 Sep 2;10(9):2291. doi: 10.3390/cells10092291 (PMC8465596; doi:10.3390/cells10092291)
Supplement: Supplementary file 1 [file cells-10-02291-s001.zip › cells-1329504- Table S2 IPA analysis network list (top 10).pdf]

Table S1. IPA analysis network list (top 10)

ID	miRNAs in Network	mRNAs in Network	Score	Focus Molecules	Top Diseases and Functions
1	miR-125aq-3p, miR-128-3p,miR-17-5p,mir-21-5p,miR342-3p,miR-487-3p,let-7a-5p,miR-125b-1-3p,miR-130a-3p,miR-18a-5p,miR-218-5p,miR-377-5p,miR-543-3p,miR-126a-3p,miR-132-3p,mir193a-3p,miR292b-5p,miR382-5p,miR-7a-5p,miR-100-5p,miR126a-5p, miR-150-5p,miR19b-3p,miR330-3pmiR-433-3p,niR-92a-3p	Akt, Insulin, Smad2/3, CG,Tgf beta, Creb,MAP2K1/2,VEGF	52	26	Cancer, Organism injury and Abnormalities, Reproductive system Disease
2	miR-218-1-3p,miR-340-5p,miR409-3p,miR-495-3p,miR-3065-5p,miR-34c-3p,miR-452-5p,miR142-5p,miR-335-3p,miR369-3p,miR-501-3p,miR-151-3p,miR-374b-5p,miR493-5p,miR-7-1-3p	AGO2,GMFB,LIG4,MRPS36,TNF, AGO3,HIGD1A, LXN,NHEJ1,UBE2W,CDH1,HNRNPA1, RP11_176H81,XKR4,GABRA4,IGFL1,TGFBR2,	25	15	Cell-To-Cell Signaling and Abnormalities, Reproductive system Disease
3	Let-7i-3p,miR-29b-1-5p,miR-515,miR-548f-3p,miR1285-3p.miR485-3p,miR-517a-3p,mir-618,LET-7E-3P,Mir-182-5P,miR-486-5p,miR-542-3p,let-7f-2-3p, miR-183-5p,miR-504-5p	AK3, CMPK1,miR-515,RAB27B,TP53INP1,C1orf131,CNMD,let-7,RPL23A,ZMAT3,C2orf88,DBI,NDUFA4L2,SKIL ZNF302,CAMK2N1,FABP2,miR-548,PLAGL1,TP53	22	14	Cancer, Organismal Injury and Abnormalities, Reproductive System Disease
4	miR-103-3p,miR-145-5p,miR214-3p,miR-548-3p,miR-10399-5p,miR-154-3p,miR-296-3p,mir-135a-5p,miR-188-3p,miR-3194-3p,miR-145-3p,miR212-5p,miR-339-5p	APEX1,CSNK1A1,IKZF2,BATF3,DDC,JUNB,C20orf197,FAM120AOS, METTL7A,NIF3L1,TGFRAP1,ZNF667,CEP57L1,GFI1,PKIA,tretinoin	20	13	Cell Morphology, Infectious Disease, Organismal Development
5	miR-147,miR22-5p,miR3909,miR766-3p,miR148a-3p,miR-223-3p,miR-545-5p,miR-154-5p,miR377-3p,miR-548o-3p,miR-197-3p,miR378a-5p,miR-574-3p	ADORA2B,FAM169A,IFIT2,TCIM,ZNF233,CCDC180,FAM9C,IKZF2,PIK3IP1,TMEM100,ZNF550,CD28,FEZ2,IL6,R RNF146,XKR4,ZNF772,EPDR1,GMFB,LINC01553,RSPO3,ZBTB37	20	13	Inflammatory Disease, Inflammatory Response, Organismal injury and Abnormalities
6	miR-138-5p,miR23a-5p,miR381-3p,miR-592,miR-	AR,CCRB,KLLN,MYoZ2,TMEM68,	20	13	

	140-5p,miR28-3p,miR-508-3p,miR18a-3p,miR-514-3p,miR-874-5p,miR-211-3p,miR-3617-5p,miR519a-3p	C1orf147,DTX4,LINC01553,miR641,POMZP3,TNF,C1orf185, ENPP5,LXN,miR-290,PRRG4,TRUB1,CAPSL,ENTPD5,LYRM1,MYD88,SMIM15			
7	miR-1255b-5p,miR-3913-5p, miR-532-3p,miR-22-3p,miR-421-3p,miR542-5p,miR-3118,miR4423-3p,miR-548h-5p,miR-1180-3p,miR344a-5p,miR483-5p,miR590-3p	ARL17A/ARL17B,DERA,ERBB2,JPT1,C5orf24,DOK6,GSG1L,KIAA0087,PTTG1IP,XPO4,CHST9,DTX4,HOXA4,MGARP,RPS15,YWHAZ,CYLC2,EGFR,HSH2D,STMN3	20	13	Cancer, Organismal Injury and Abnormalities, Renal and urological Disease
8	miR-155-3p,miR-4723-5p,miR-548v,let-7a-2-3p,miR-192-5p,miR489-3p,miR-509-5p,mir-570-3p,mir-346,miR493-3p,miR-511-5p,miR-450a-5p,miR-503-5p	ANGPTL7,CYBRD1,L2HGDH,PRR9,ZBTB37,C18orf25,FAM9C,RAB31,ZNF256,C1D,GABRA4,LITAF,MYBL1,SLC25A36,ZNF420,CXCL10,IL13,METTL21A,miR-548,NFIB,TGFB1	20	13	Cardiovascular System development and Function, Cellular Growth and proliferation
9	miR-1247-5p,mir-144-3p,miR-424-3p,mir-708-5p,mir-132-5p,mir328-3p,miR-486-3p,mir-133a-5p,miR329-3p,miR-507,miR-143-5p,miR344d-3p,miR-513a-3p	C1QTNF6,FOS,HIF1A,PIGA,TM4SF1,C2orf88,GIT2,KIAA1217,NFATC2IP,PROK2,TNFSF11,CCNG2,GLRX,KPNA5,OGG1,RAB36,ZNF667,FMR1,GRIN1,MBD3,PDK4,TFF3	20	13	Cell-to Cell Signaling and Interaction, cellular Development, Nervous System Development and Function
10	miR-152-5p,miR-3173-5p, mir-548e-5p,miR-156-5p,miR3585-5p,miR-642a-5p,miR-1268a,miR-185-5p,miR-365-1-5p,miR-1301-3p,miR-30c-1-3p,mir-513c-5p	ARL3,FAM169A,JUN,PCDHAC2,RHOA,TMEM100,C18orf25,GPRB84,LPAR5,PDE6B,RSPO3,TMIE,CARD18,HMOX1,OMG,RAB31,SLC5A8,TNFRS10B,DGCR2,HRAS,PALM2AKAP2,RELL1,SSBP3-AS1	18	12	Cell Death and survival, Post-Translation Modification, Protein Degradation
